# Supplementary figures and images for: Social reputation influences on liking and willingness-to-pay for artworks: A multimethod design investigating choice behavior along with physiological measures and motivational factors
Source: PLoS One. 2022 Apr 20;17(4):e0266020. doi: 10.1371/journal.pone.0266020 (PMC9020698; doi:10.1371/journal.pone.0266020)

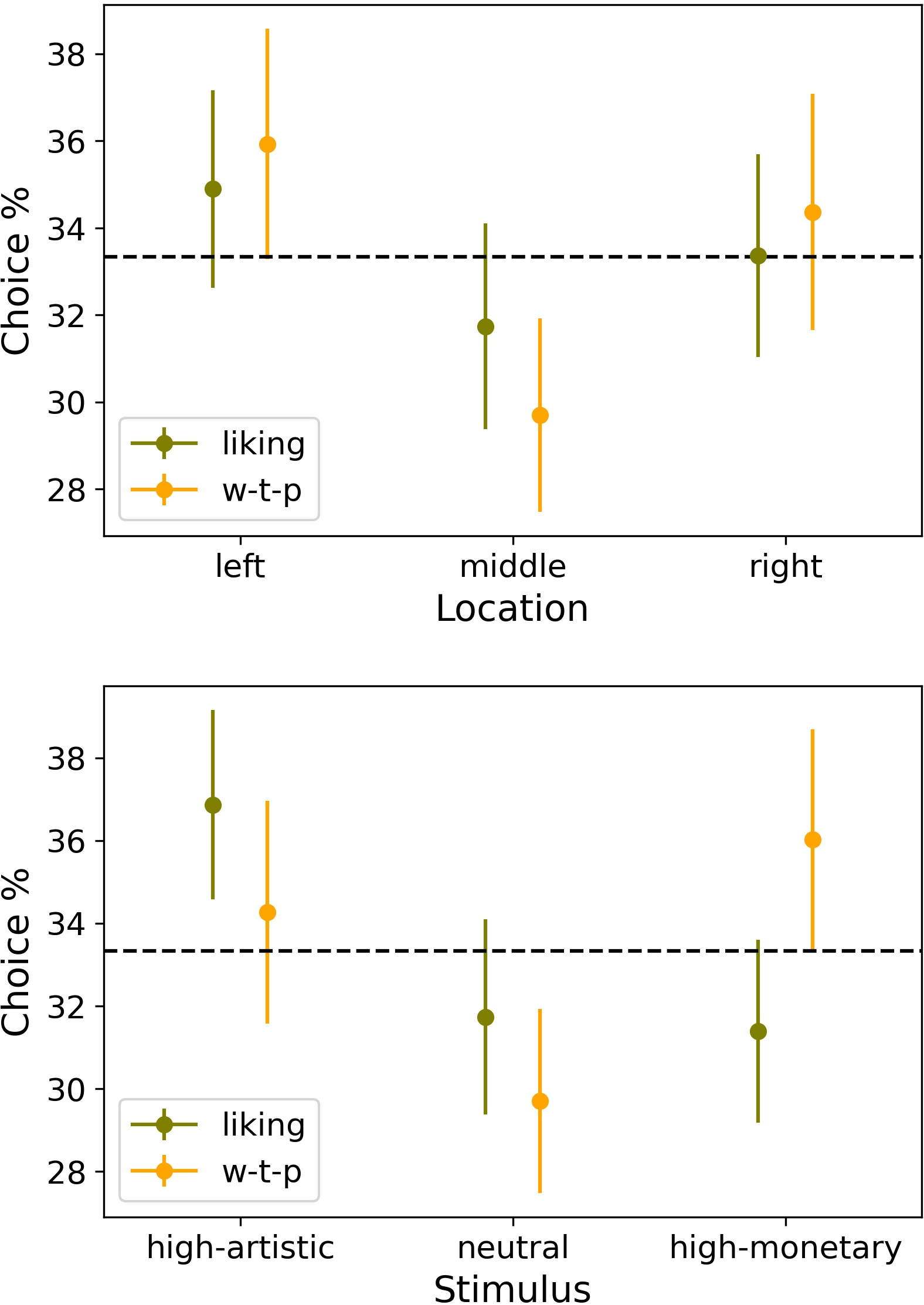

Supplement: S1 Fig — A. Descriptive analysis of position and value position. The dashed horizontal lines show chance (1/3≈33.3%) choice. Error-bars represent 2 standard errors of the mean. A. Effect shows the average choice percentage for the three locations (x-axis) separated by choice type (see legend). B. Descriptive analysis of position and value position. The dashed horizontal lines show chance (1/3≈33.3%) choice. Error-bars represent 2 standard errors of the mean. B Effect shows the average choice percentage for the three stimulus types (x-axis) separated by choice type (see legend). (TIF) [file pone.0266020.s001.tif]

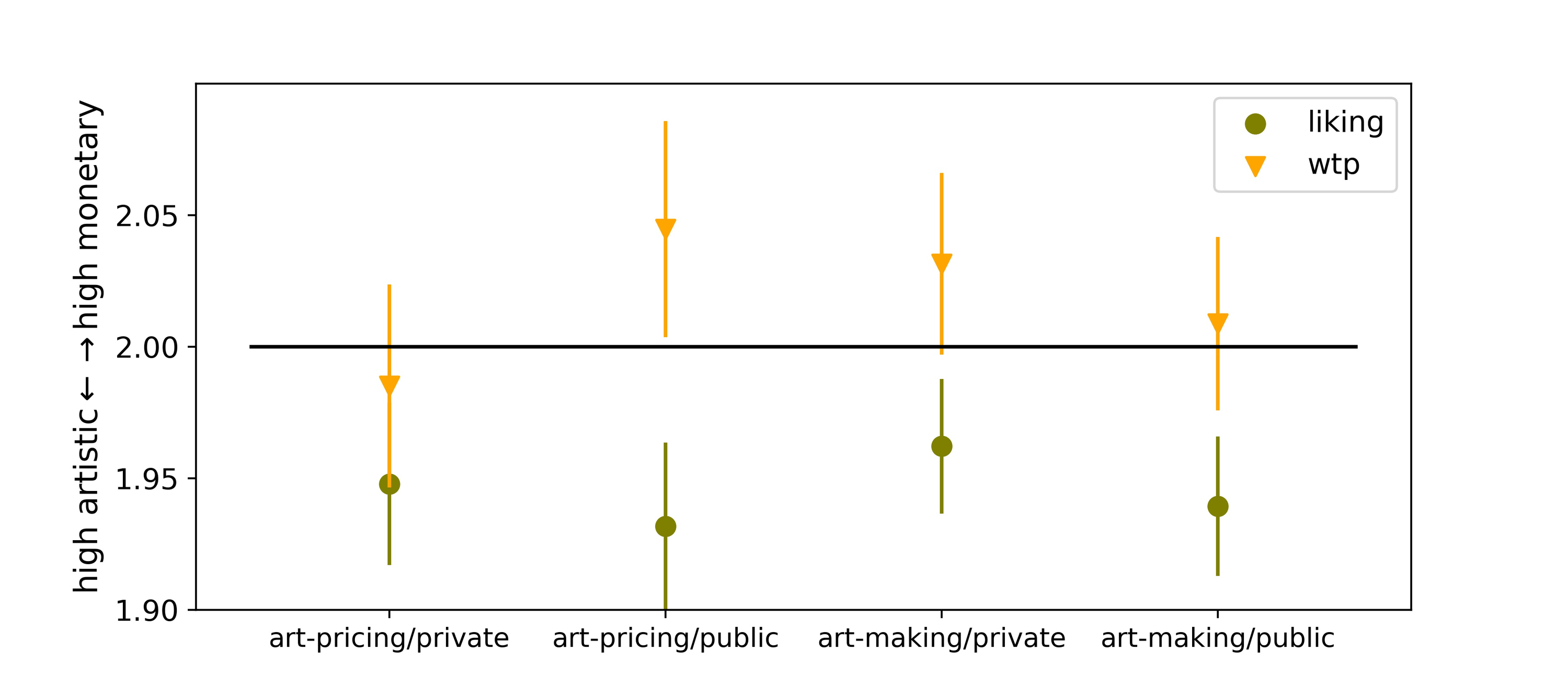

Supplement: S2 Fig — (TIF) [file pone.0266020.s002.tif]

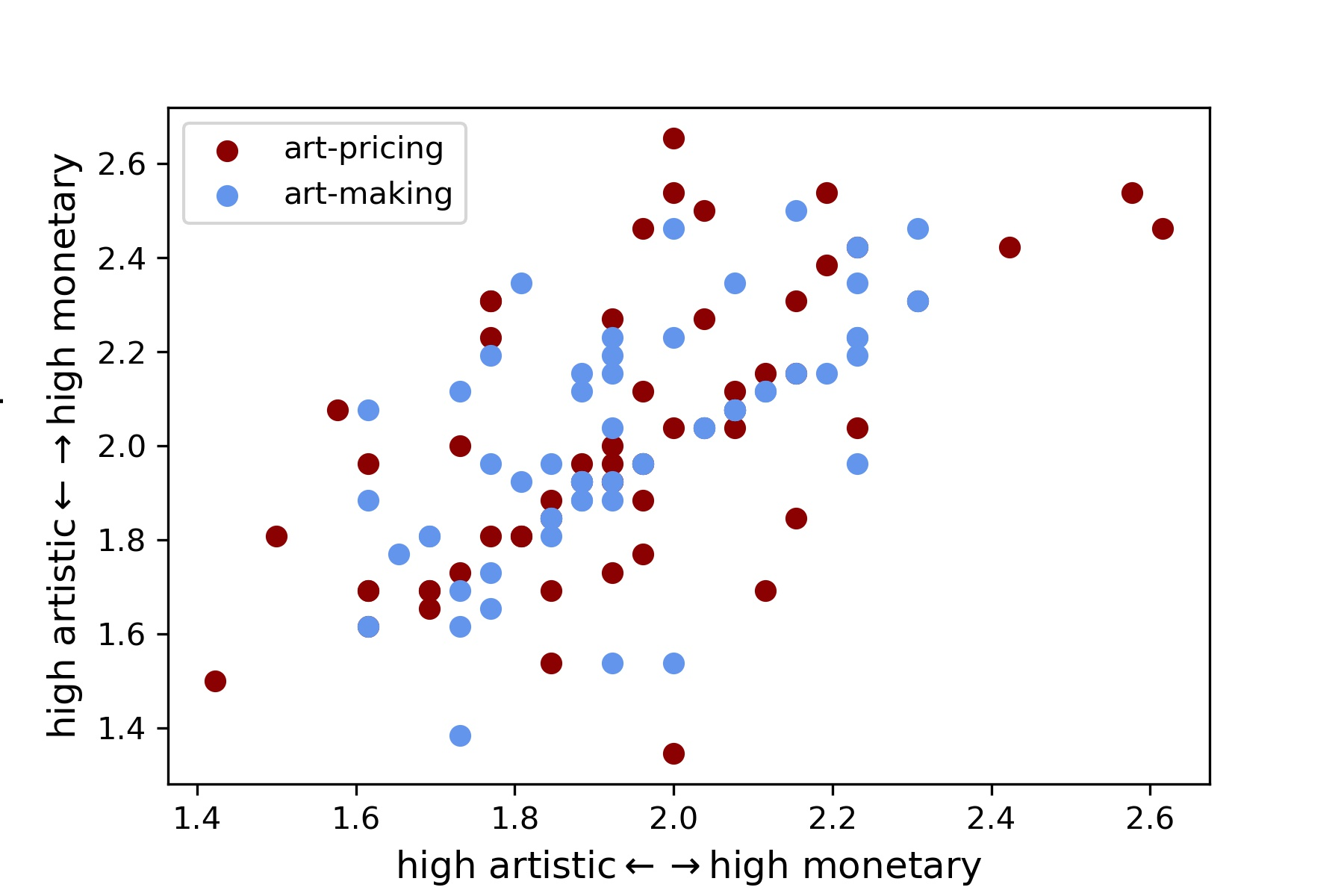

Supplement: S3 Fig — Each dot represents the average preference for stimulus type (y-axis) for a participant for liking and willingness-to-pay choices (x-axis). (TIF) [file pone.0266020.s003.tif]

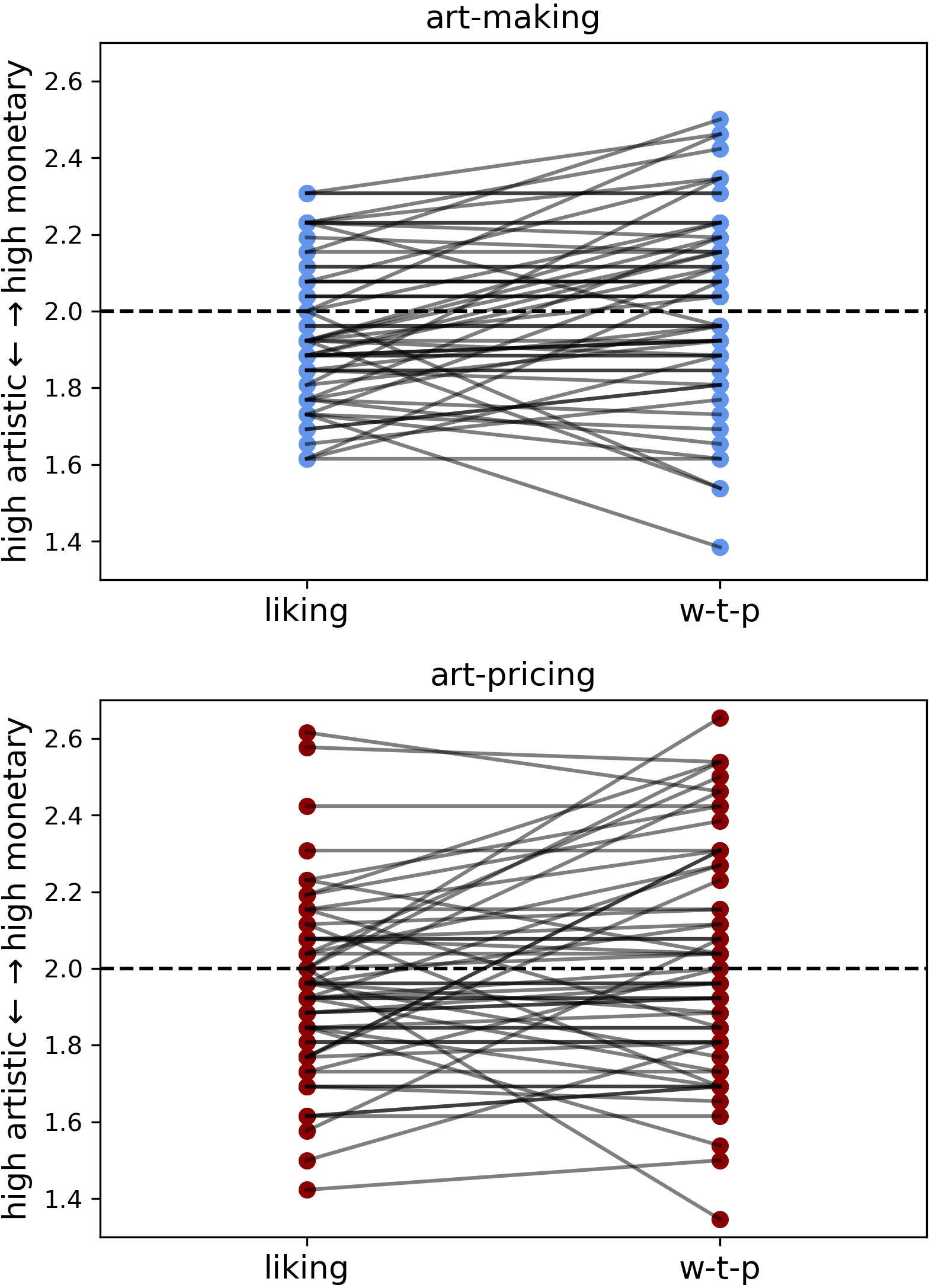

Supplement: S4 Fig — A. Between subject variability reported for both choice types. Gray lines connect dots within participants. The dashed line shows the—on average—neutral choice. A. Art-making group. B. Between subject variability reported for both choice types. Gray lines connect dots within participants. The dashed line shows the—on average—neutral choice. B. Art-pricing group. (TIF) [file pone.0266020.s004.tif]

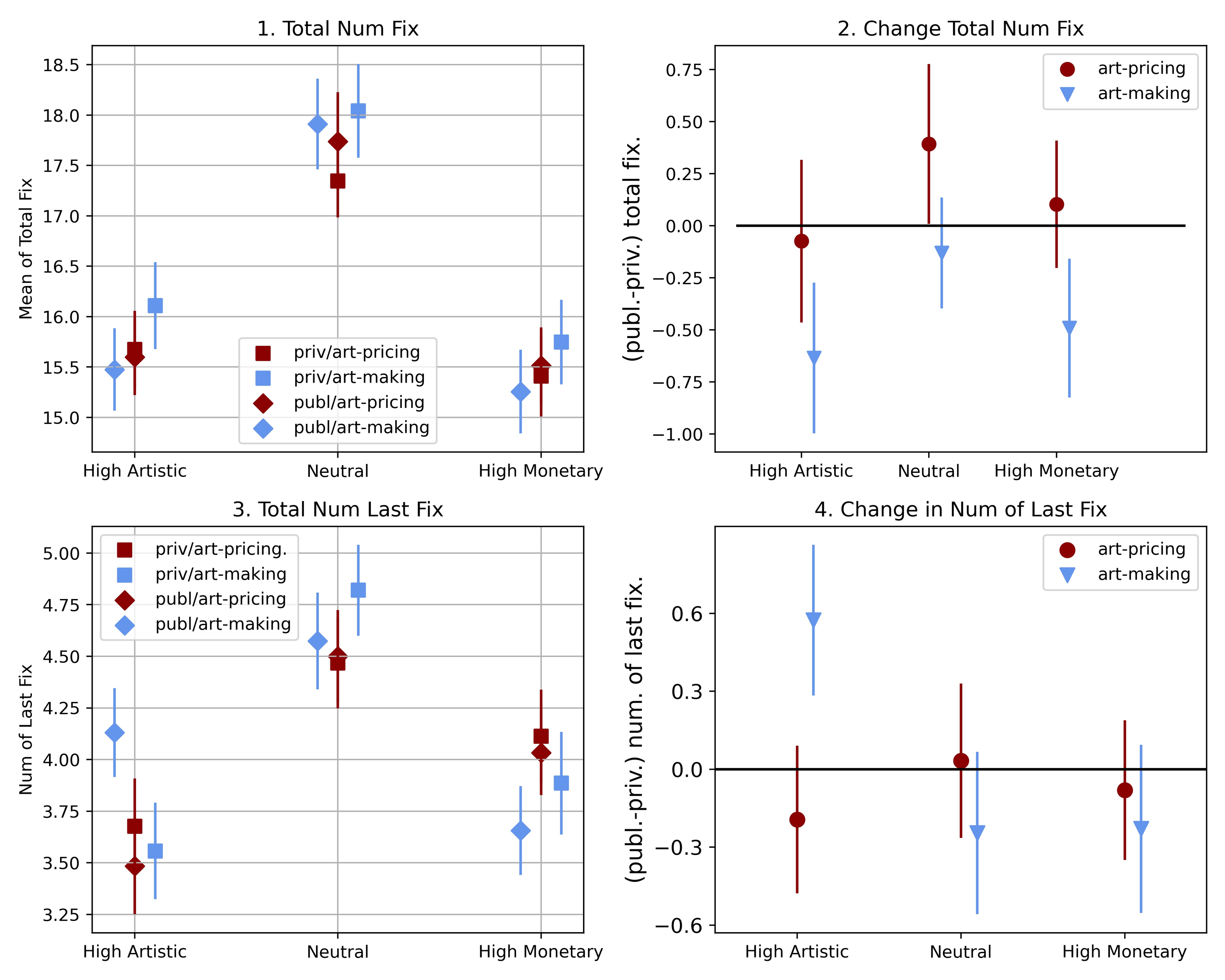

Supplement: S5 Fig — Left: Total number of fixations and last fixations in the different conditions. Right: Change in gaze behavior between the two audience conditions calculated as public minus private. Upper figures show results for total number of fixations. Figures below for last fixations. (TIF) [file pone.0266020.s005.tif]
