# Supplementary material for: Social reputation influences on liking and willingness-to-pay for artworks: A multimethod design investigating choice behavior along with physiological measures and motivational factors
Source: PLoS One. 2022 Apr 20;17(4):e0266020. doi: 10.1371/journal.pone.0266020 (PMC9020698; doi:10.1371/journal.pone.0266020)
Supplement: S1 Table — (PDF) [file pone.0266020.s006.pdf]

S1 Table. List of used artworks and assignment of the artworks in the sets.

No	Set.	Artist	Year	Title	Epoch/Style
1.1	1	Blanchard, María	1916	Composición cubista	Kubismus
1.2	1	Valmier, Georges	1928	The Tulips	Kubismus
1.3	1	Gleizes, Albert	1916	On a Sailboat	Kubismus
4	2	Delaunay, Robert	1912	Simultanous Windows	Orphischer Kubismus, Synchromismus
5	2	Kupka, František	1912	untitled	Orphischer Kubismus, Synchromismus
6	2	Severini, Gino	unknown	Ballerina bow sea	Orphischer Kubismus, Synchromismus
7	3	O`Keeffe, Georgia	unknown	unknown	Präzisionismus
8	3	O`Keeffe, Georgia	1921	Blue and Green Music	Präzisionismus
9	3	Sheeler, Charles	1917	Flower Forms	Präzisionismus
10	4	Lewis, Percy Wyndham	1914-15	untitled	Vortizismus
11	4	Saunders, Helen	1914-15	Vorticist Composition Black and Khaki	Vortizismus
12	4	Wadsworth, Edward	1915	Abstract Composition	Vortizismus
13	5	Lazar Markovich Lissitzky (El Lissitzky)	1922	Composition	Suprematismus - Konstruktivismus
14	5	Lazar Markovich Lissitzky (El Lissitzky)	1922	Proun 19D	Suprematismus - Konstruktivismus
15	5	Moholy-Nagy, Lazlo	1925	Composition A XXI	Suprematismus - Konstruktivismus
16	6	Malewitsch, Kasimir	unknown	unknown	Suprematismus - Konstruktivismus
17	6	Moholy-Nagy, Lazlo	1924	A II	Suprematismus - Konstruktivismus
18	6	Moholy-Nagy, Lazlo	1922	lis	Suprematismus - Konstruktivismus
19	7	Mondrian, Piet	unknown	unknown	De Stijl
20	7	Mondrian, Piet	1941-42	New York	De Stijl
21	7	Doesburg van, Theo	1924	Kontra-Komposition V	De Stijl
22	8	Klee, Paul	1922	Red Balloon	Bauhaus
23	8	Klee, Paul	1929	Stufen	Bauhaus
24	8	Klee, Paul	1931	Aufgehender Stern	Bauhaus
25	9	Kandinsky, Wassily	1933	Soft Harshness	Bauhaus
26	9	Albers, Josef	1936	Prismatic II	Geometrische Abstraktion
27	9	Gallatin, Albert Eugene	1937-38	Room Space	Geometrische Abstraktion
28	10	Kline, Franz	1956	Mahoning	Abstrakter Expressionismus
29	10	Kline, Franz	1958	American	Abstrakter Expressionismus
30	10	Kooning de, Willem	1948	Black Friday	Abstrakter Expressionismus
31	11	Debré, Olivier	1955	Cliffs	Tachismus
32	11	Soulages, Pierre	1953	Painting, 195 x 130 cm, May 1953	Tachismus
33	11	Soulages, Pierre	1962	"3.Mai 1962"	Tachismus
34	12	Johns, Jasper	1960s	Decoy	Neo-Dada, Nouveau Realism
35	12	Johns, Jasper	1963	Land`s End	Neo-Dada, Nouveau Realism
36	12	Rauschenberg, Robert	1956	Gloria	Neo-Dada, Nouveau Realism
37	13	Calder, Alexander	unknown	unknown	Modern art
38	13	Calder, Alexander	unknown	unknown	Modern art
39	13	Calder, Alexander	unknown	unknown	Modern art
40	14	Macke, August	1912	Coloured Composition (Hommage à Johann Sebastin Bach)	Expressionismus
41	14	Morgner, Wilhelm	1912	Astral Komposition	Expressionismus
42	14	Kandinsky, Wassily	1913	Sketch I for Painting with White Border, Moscow	Expressionismus
43	15	Picabia, Francis	unknown		Kubismus
44	15	Picabia, Francis	1914	Comic Wedlock	Kubismus
45	15	Picabia, Francis	1914	This Has to Do with Me	Kubismus
46	16	Demuth, Charles	1920	Machinery	Präzisionismus
47	16	Picabia, Francis	1915	Very rare Picture in the World	Präzisionismus
48	16	Schamberg, Morton	1916	Painting VIII, Mechanical Abstraction	Präzisionismus
