# Supplementary material for: Social reputation influences on liking and willingness-to-pay for artworks: A multimethod design investigating choice behavior along with physiological measures and motivational factors
Source: PLoS One. 2022 Apr 20;17(4):e0266020. doi: 10.1371/journal.pone.0266020 (PMC9020698; doi:10.1371/journal.pone.0266020)
Supplement: S3 Table — Independent variable between-participant factor audience type and within-participant factor public vs. private. Dependent variable choice type willingness-to-pay. (PDF) [file pone.0266020.s008.pdf]

S3 Table. Two-way mixed ANOVA for willingness-to-pay.

Variables	F (1,121)	p	η^2
Audience type (art-making/art-pricing experts)	0.012	.91	0.000
Within-participant variable (public/private)	1.030	.31	0.008
Interaction	4.949	**.03**	0.039

Independent variable between-participant factor audience type and within-participant factor public vs. private. Dependent variable choice type willingness-to-pay.
