# Supplementary material for: Social reputation influences on liking and willingness-to-pay for artworks: A multimethod design investigating choice behavior along with physiological measures and motivational factors
Source: PLoS One. 2022 Apr 20;17(4):e0266020. doi: 10.1371/journal.pone.0266020 (PMC9020698; doi:10.1371/journal.pone.0266020)
Supplement: S4 Table — (PDF) [file pone.0266020.s009.pdf]

S4 Table. Mean and standard deviations of total number of fixation and last fixation over all 13 trials between the condition.

within-participant factor	Private						Public					
	High artistic		Neutral		High monetary		High artistic		Neutral		High monetary	
	M	*SD*	*M*	*SD*	*M*	*SD*	*M*	*SD*	*M*	*SD*	*M*	*SD*
Total number of fixations												
Art-pricing experts	15.67	2.99	17.35	2.83	15.41	3.13	15.60	2.96	17.74	3.81	15.51	2.98
Art-making experts	16.11	3.34	18.04	3.60	15.75	3.24	15.47	3.17	17.91	3.49	15.26	3.22
Last fixations												
Art-pricing expert s	3.68	1.80	4.47	1.72	4.11	1.76	3.48	1.82	4.5	1.75	4.03	1.60
Art-making experts	3.56	1.81	4.82	1.70	3.89	1.93	4.13	1.66	4.57	1.86	3.66	1.67
