# Supplementary material for: Social reputation influences on liking and willingness-to-pay for artworks: A multimethod design investigating choice behavior along with physiological measures and motivational factors
Source: PLoS One. 2022 Apr 20;17(4):e0266020. doi: 10.1371/journal.pone.0266020 (PMC9020698; doi:10.1371/journal.pone.0266020)
Supplement: S5 Table — (PDF) [file pone.0266020.s010.pdf]

S5 Table. Mean of beta and standard deviations of total amount of fixations between the two audience type conditions and for both choice types, liking and willingness-to-pay (wtp).

Choices	between-participant factor	*M*	*SD*
liking	Art-pricing experts	0.51	0.26
	Art-making experts	0.46	0.30
wtp	Art-pricing experts	0.46	0.26
	Art-making experts	0.41	0.28
