# Supplementary material for: Social reputation influences on liking and willingness-to-pay for artworks: A multimethod design investigating choice behavior along with physiological measures and motivational factors
Source: PLoS One. 2022 Apr 20;17(4):e0266020. doi: 10.1371/journal.pone.0266020 (PMC9020698; doi:10.1371/journal.pone.0266020)
Supplement: S6 Table — Included predictors were BIS, BAS (Drive + Fun), LSAS-anxiety, and between-participant condition. (PDF) [file pone.0266020.s011.pdf]

S6 Table. Linear regression model for predicting liking versus willingness-to-pay (wtp) choices.

Liking vs. wtp	*B*	*SE*	95% CI	r^2	*t*	*p*
Intercept	-0.30	0.19	[-0.67,0.08]	0.08	-1.56	.12
BIS	0.01	0.05	[-0.09,0.12]	0.08	0.27	.79
BAS (Drive + Fun)	0.07	0.02	[0.02,0.12]	0.08	2.78	**<.01**
LSAS Anxiety	-0.00	0.00	[-0.01,0.00]	0.08	-1.22	.22
art-making/art-pricing experts	-0.01	0.04	[-0.09,0.06]	0.08	-0.39	.70

Included predictors were BIS, BAS (Drive + Fun), LSAS-anxiety, and between-participant condition.
