# Supplementary material for: Social reputation influences on liking and willingness-to-pay for artworks: A multimethod design investigating choice behavior along with physiological measures and motivational factors
Source: PLoS One. 2022 Apr 20;17(4):e0266020. doi: 10.1371/journal.pone.0266020 (PMC9020698; doi:10.1371/journal.pone.0266020)
Supplement: S1 File — (PDF) [file pone.0266020.s012.pdf]

***S1 File. Cover Story (in German).***

*„Die folgende Studie wurde in Kooperation mit dem bekannten österreichischen Auktionshaus Wittelsburg und der Akademie der Künste Wien entwickelt. Die Forschungspartner wollen, dass wir erforschen, welche visuellen Elemente in den Kunstwerken, auf das Gefallen, das Bietverhalten und die Kaufentscheidung Einfluss nehmen können. (weiter mit Leertaste)*

*Für dieses Experiment haben wir in enger Zusammenarbeit mit beiden Kooperationspartnern ein außergewöhnliches Set von Kunstwerken derselben Künstler zusammenstellen können, die sich systematisch hinsichtlich des künstlerischen Werts, im Sinne kunstwissenschaftlicher Analysen, und ihrem monetären Wert, den sie bei Auktionen erzielt haben, unterscheiden. Sie werden entsprechend dieser Zusammenstellung immer drei Kunstwerke nebeneinander sehen. Sie haben jeweils 20 Sekunden Zeit die Kunstwerke zu betrachten. Währenddessen brauchen Sie nichts tun, es geht dann automatisch weiter. Bitte versuchen Sie sich die Bilder gut einzuprägen. (weiter mit Leertaste)*

*Durch die Kooperation mit dem Auktionshaus Wittelsburg und mit der Akademie der Künste, war es möglich Kunstwerke zu finden, die sich in ihrem künstlerischen Wert und in ihrem monetären Wert unterscheiden bzw. ähnlich sind. Künstlerischer Wert bedeutet, dass dieses Kunstwerk unter Experten einen besonderen ästhetischen, historischen, wissenschaftlichen oder gesellschaftlichen Wert für vergangene, gegenwärtige und zukünftige Generationen hat. Monetärer Wert ist der Preis, den das Kunstwerk bei der aktuell letzten Auktion erzielt hat. (weiter mit Leertaste)*

*Für die gemeinsame Studie wurden speziell Kunstwerke ausgewählt, die sich genau hinsichtlich ihres künstlerischen und monetären Werts unterscheiden oder eben ähneln. Sie werden daher 3er Sets von visuell sehr ähnlichen Kunstwerken sehen (gleicher Stil, gleicher*

*Künstler), die entweder einen: hohen künstlerischen Wert, jedoch niedrigen monetären Wert; niedrigen künstlerischen Wert, jedoch hohen monetären Wert; ähnlich hohen künstlerischen und monetären Wert haben. Sie erkennen an der Beschriftung unterhalb der Kunstwerke, wie die Wert-Verhältnisse zueinanderstehen.“*

**Separation in between and within participants.**

*„Um das Verhalten und ihre Bewertungen genau untersuchen zu können, wurden wir von den Fachleuten des Auktionshauses Wittelsburg/Fachleuten der Akademie der Künste gebeten, einen der beiden Blöcke per Kamera aufzunehmen, um diese dann genauer auswerten zu können. Sie werden darüber informiert, welcher der beiden Blöcke mitgefilmt wird.*

*Der andere Block dient der Universität Wien zur Datenauswertung und unterliegt den strengen Anonymitätskriterien der Universität Wien. Unser Interesse besteht insbesondere darin, Zusammenhängen von physiologischen Maßen (u.a. Hormonlevels), visuelle Kunstwahrnehmung und Evaluation zu untersuchen. Wir bitten Sie in beiden Blöcken möglichst spontane Entscheidungen abzugeben.“*
