# Supplementary material for: Social reputation influences on liking and willingness-to-pay for artworks: A multimethod design investigating choice behavior along with physiological measures and motivational factors
Source: PLoS One. 2022 Apr 20;17(4):e0266020. doi: 10.1371/journal.pone.0266020 (PMC9020698; doi:10.1371/journal.pone.0266020)
Supplement: S2 File — (PDF) [file pone.0266020.s013.pdf]

S2 File. Cover Story (in English).

"The following study has been developed in cooperation with the well-known Austrian auction house Wittelsburg and the Academy of Arts Vienna. The research partners want us to explore which visual elements in artworks, can influence liking, bidding behavior and buying decisions. (continue with spacebar)

In close collaboration with both cooperation partner, we have for this experiment been able to compile an extraordinary set of artworks by the same artists, which differ systematically in terms of artistic value, in the sense of art-scientific analyses, and their monetary value, which they have achieved at auctions. According to this compilation, you will always see three artworks next to each other. You will have 20 seconds time to look at each of the sets of artworks. During this time, you do not need to do anything but look at the images, it will continue automatically. Please try to memorize the pictures. (continue with space bar)

Due to the cooperation with the auction house Wittelsburg and with the Academy of Arts, it was possible to find artworks, which are different or similar in their artistic value and in their monetary value. Artistic value means that this work of art has, among experts, a special aesthetic, historical, scientific, or social value for past, present, and future generations. Monetary value is the price the artwork received at the current last auction. (continue with spacebar)

For the joint study, artworks were specifically selected that differ exactly in terms of their artistic and monetary value or are similar. You will therefore see sets of 3 visually very similar artworks (same style, same artist) that have either: high artistic value but low monetary value; low artistic value but high monetary value; similar high artistic and monetary value. You can tell by the labeling below the artworks how the value ratios relate to each other."

Separation in between and within participants.

"In order to be able to examine the behavior and their valuations in detail, we were asked by the experts of the auction house Wittelsburg/experts of the Academy of Arts, to record one of the two blocks by camera in order to be able to evaluate them more precisely for future investigations. You will be informed which of the two blocks will be filmed.

The other block will be used by the University of Vienna for data analysis and will be subject to the strict anonymity regulations of the University of Vienna. We are particularly interested in investigating correlations between physiological measures (including hormone levels), visual art perception and evaluation. We ask you to give as spontaneous choices as possible in both blocks."
