# Supplementary material for: Social reputation influences on liking and willingness-to-pay for artworks: A multimethod design investigating choice behavior along with physiological measures and motivational factors
Source: PLoS One. 2022 Apr 20;17(4):e0266020. doi: 10.1371/journal.pone.0266020 (PMC9020698; doi:10.1371/journal.pone.0266020)
Supplement: Attachment — Submitted filename: Response to Reviewers.docx [file pone.0266020.s015.docx]

**Response to Reviewers & Editor**

We are pleased that with our last revision we could address mostly all issues raised, included all the great feedback from the Editor and the Reviewers. We read, again, the manuscript thoroughly and corrected the minor changes suggested by Reviewer 1 (marked in red).

Thank you very much for this great collaboration. We are looking forward to further working with you all towards a successful publication!

The authors

**Reviewer #1:**

*I have now read the revised version of the manuscript. The authors have done a good job with the revisions and have largely addressed my concerns. The additional introductory information has provided a stronger rationale for the research and a fuller explanation of its novelty and importance.*

**Reply:**	Thank you very much for reviewing the revised manuscript. We are pleased that our rationale could been strengthened within the revised manuscript, and we want to thank the reviewer for all the valuable feedback that has led to the new intro. We are also pleased that we have been able to address most of the issues to the reviewer’s satisfaction.

*Line 517-522. Although I am largely persuaded by the data (line 517-522) that position did not affect choice, I do not think it can be entirely discounted. Although the artworks were randomly allocated to position, the value labelling of an artwork in the left and right positions (Left, high-artistic: Right, high-monetary) remained the same throughout the task. Position was confounded with the value labelling of the artworks and ideally the position of the value labels should have been counterbalanced. This is relevant because, in addition to a preference for items in the center, there are also perceptual asymmetries, with a body of work finding that visual information on the left-side carries greater weight/importance when making a perceptual judgement (e.g. Nicholls et al. 1999,* [*https://doi.org/10.1016/S0028-3932(98)00074-8)*](https://doi.org/10.1016/S0028-3932(98)00074-8))*. While I think the data suggest an effect of value labelling for particular positions may not have been a problem, it is not certain. Consequently, I think the statement (line 770-773) that ‘an effect of position could be ruled out’ is too strong. This should be attenuated to something like ‘The effect of position with respect to choice may be unlikely…’*

**Reply:**	Thank you very much for reviewing this again. We corrected in line (now) 777-780 the wording. We also want to refer to the Methods section that the labelling was also counterbalanced between participants. Even the coin and brush sign (left/right) under each image was counterbalanced between participants. However, it is true, that labelling was not changed anymore within the same participant (we added this description in the text, see lines 386-389, and in the Figure, see line 203-208). The reasoning is that we did want the participants to remember the label weights, in order to avoid repetitive eye-movement patterns to the labels below the images. This would have been distractive for focusing on the choice task and for the eye-movement pattern analysis.

*Line 143 – ‘greatly less’, should this be ‘less’.*

**Reply:**	Thank you. We deleted the word ‘greatly’.  *Line 146, ‘have shown to be more vulnerability to reputation influences’ should be ‘…..more vulnerable….’*

**Reply:**	Corrected.  *Line 765 “landing position, see for further reading [80, 97-99])”. Including the following reference in this list is appropriate as it examined the effects of position on the choice of artworks and also used triplets of similar artworks while measuring eye tracking (Kreplin et al. 2014,* [*https://doi.org/10.1016/j.actpsy.2014.08.003*](https://doi.org/10.1016/j.actpsy.2014.08.003)*).*

**Reply:**	Thank you. We added the additional reference.  *Congratulations on an interesting study.*

**Reply:**	We would like to express our sincere appreciation for the review of our manuscript, the rich input, and feedback.

**Reviewer #2:**

*Reviewer #2: All the comments have been adressed. The authors did a good job.*

**Reply:**	We are very happy that we could address all the interesting points, suggestions, and issues raised made by the reviewer. We would like to thank the reviewer very much for all the effort, also checking the reviewed manuscript.
